# A scoping review of the incentives for promoting the adoption of agroecological practices and outcomes among rice farmers in Vietnam

**DOI:** 10.1371/journal.pone.0321029

**Published:** 2025-04-25

**Authors:** Sylvester Ogutu, Jonathan Mockshell, Thai Minh, Roseline Remans

**Affiliations:** 1 International Center for Tropical Agriculture (CIAT), Kampala, Uganda; 2 International Center for Tropical Agriculture (CIAT), Cali, Colombia; 3 International Water Management Institute, Accra, Ghana; 4 Bioversity International, Montpellier, France; Canakkale Onsekiz Mart University, TÜRKIYE

## Abstract

Recent research and development efforts to achieve sustainable rice production in Vietnam have incorporated agroecological principles and practices. These efforts have come as a result of increasing pressure on food systems to achieve global food security. Growing food demand, rising population, climate change, and natural resources degradation, make it necessary to transform the current production systems towards more sustainable models. Agroecology is being promoted as one of the pathways to transition toward sustainable food production, with broad adoption requiring incentives for farmers. Using the Preferred Reporting Items for Systematic Reviews and Meta-Analyses extension for Scoping Reviews guidelines, we conducted a scoping review of 120 articles to understand the incentives applied for promoting agroecological practices, whether and how the incentives promote the adoption of agroecological practices, and their relationship with economic, environmental, and social outcomes among rice farmers in Vietnam. Such in-depth reviews of the role of incentives in the agroecological transition are scarce. Results show that in about 60% of the articles, incentives led to adoption, outcomes (e.g., yield and income gains), or both, suggesting the importance and common use of incentives among rice farmers in Vietnam. Four types of incentives used were market, non-market, regulatory, and cross-compliance. These incentives directly or indirectly influenced outcomes through the adoption of agroecological practices. Market incentives (33%) were relatively more important for directly influencing outcomes, while non-market incentives (50%) were more important for indirectly influencing outcomes. Market, non-market, and regulatory incentives were more associated with the adoption of new agronomic practices, such as farm diversification, improved varieties, and organic agriculture than with other practices, while cross-compliance incentives were more highly associated with adoption of agroforestry. Generally, the incentives used were more associated with economic outcomes (56%), than with environmental (31%), and social (13%) outcomes. Overall, the results show that incentives influence outcomes differently, and a combination of different incentives is required to achieve holistic outcomes.

## Introduction

Growing food demand, rising population, deforestation, biodiversity loss, climate change, and natural resource degradation are increasing the pressure on global food systems to transform into sustainable food systems in order to achieve global food security [1,2]. Sustainable food systems―which are achieved through practices such as farm diversification, crop rotation, zero tillage, agroforestry, integrated pest management, and crop–livestock integration―ensure good stewardship of the natural resources that farms rely on. Sustainable food systems and policies aim to increase farm productivity while maintaining environmental sustainability [2].

Agroecology is increasingly seen as an effective means to transition to sustainable food systems [3,4]. Agroecology is defined as the application of ecological concepts and principles in farming to optimize interactions between plants, animals, humans, and the environment while considering the social aspects needed for a sustainable and inclusive food system. It entails 13 principles including recycling, input reduction, soil health, animal health, biodiversity, synergy, economic diversification, knowledge co-creation, social values and diets, fairness, connectivity, land, and natural resource governance, and participation (see Table 1) [4,5]. Agroecology can play a critical role in protecting the ecosystem by ensuring more efficient use of natural resources, strengthening the capacity to adapt to climate change and climate variability, and increasing productivity [2,6].

**Table 1:** The 13 principles of agroecology (short name presented in bold font).

Principle 1: **Recycling**, including closing nutrient and biomass resource cycles
Principle 2: **Input reduction** through reducing or eliminating use of chemicals or environmentally harmful inputs
Principle 3: Enhancing **soil health** through improving soil biodiversity and use of organic material
Principle 4: Ensuring **animal health** and welfare
Principle 5: Enhancing **biodiversity** at field, farm and landscape scales
Principle 6: Enhancing **synergies** across agronomic or environmental outcomes by strengthening ecological interactions and processes
Principle 7: **Economic diversification**, to provide greater financial security to farmers
Principle 8: **Co-creation of knowledge**, including empowering farmers as data owners and encouraging farmer-to-farmer knowledge exchange
Principle 9: Respecting **social values and diets**, including enhancing social cohesion and putting community-driven priorities at the center of decision-making
Principle 10: Fostering **fairness** including ensuring that all food system actors have respectable and sustainable livelihoods centered on fair trade, safe and dignified labor conditions and fair intellectual property rights
Principle 11: Increasing **connectivity** between producers and consumers by promoting local markets and short distribution networks
Principle 12: Strengthening local **land and natural resource governance**, including recognizing and empowering smallholders and indigenous peoples as sustainable land and natural resource managers
Principle 13: Encourage and facilitate **participation** of food producers and consumers in decision making, including women, youth and minority groups

Source: Adapted from Wezel et al. (2020) and Jones et al. (2022).

The adoption of agroecological practices usually requires significant effort from farmers, the support of governments and public–private partnerships at the national and local levels, capital investments, and incentives [2,7]. Incentives, such as subsidies, price premiums, technical support, and regulations can encourage farmers to protect ecosystem services (e.g., soil, water, and forest resources). They can promote the adoption of agroecological practices, while also improving agricultural outcomes, such as productivity, profitability, and farm income [8]. Although incentives are important, the adoption of agroecological practices may also depend on other factors, such as the farmers’, farm, and contextual characteristics [9–12].

In this article, we study the incentives for agroecological transition among rice farmers in Vietnam. We conduct a context-, country- and value chain-specific review of the literature, based on the assumption that agroecological transition or sustainability issues are often landscape- and context-specific [4]. We focus on rice farming in Vietnam since recent public and private sector efforts to achieve sustainability in rice production in Vietnam have incorporated agroecological principles and practices [12,13]. These efforts include several programs, such as the “Three Reductions, Three Gains” (3R3G), “One Must Do, Five Reductions” (1M5R), “Alternate Wetting and Drying” (AWD) water management technology, and the “Large Field Model” (LFM) that promote methods to transition towards more agroecological and sustainable rice production systems (further details below) [12–15]. Vietnam is the world’s third largest exporter of rice. These exports contribute positively to livelihoods and food security, and negatively to greenhouse gas (GHG) emissions [16,17]. Reducing GHG emissions in the agriculture sector requires adoption of agroecological practices. For this to happen incentives and investments are critical. We examine farm-level practices―rather than relationships focusing on the broader principles of agroecology related to governance, value chains, and equity―as they are an important first step towards agroecological transition.

Previous studies have examined the role of incentives in mitigating climate change, encouraging the adoption of sustainable practices, conserving agrobiodiversity, and improving economic well-being. Most studies on the incentives used among rice farmers in Vietnam report positive effects of incentives on economic, environmental, or social outcomes [13,14,18–21]. Nevertheless, to our knowledge, there are no scoping reviews that synthesize the literature to provide an in-depth understanding of the incentives used to promote the adoption of agroecological practices, or on whether and how the incentives promote the adoption of agroecological practices, and their association with economic, environmental, or social outcomes among rice farmers in Vietnam. Beyond Vietnam, there are a few general reviews that examine the role of incentives for adopting sustainable agricultural practices to enable the transition towards more sustainable food systems [2,22]. However, they do not focus on rice farming or specific contexts, yet context is important in addressing agroecological issues [4]. To our knowledge, only Mockshell et al. [7] have conducted an in-depth and context-specific scoping review of incentives for agroecology, in a study of wheat value-chains in Ethiopia.

To address the gaps in the literature, we conducted a scoping review to (i) synthesize the literature and understand the incentives used to promote the adoption of agroecological practices; (ii) understand whether and how the incentives promote the adoption of agroecological practices; and (iii) understand the relationship between incentives, agroecological practices, and economic, environmental, or social outcomes among rice farmers in Vietnam. Understanding the incentive mechanism and how they promote agroecology is important to help policymakers, development practitioners, and researchers identify or tailor appropriate incentives for promoting the uptake of agroecological practices among farmers. Our review findings will ensure that only the most relevant incentives that have been proven to increase the uptake of agroecological practices among farmers will be used. This review will also help researchers identify important gaps in the literature that should be addressed by future studies.

The remainder of this article is organized as follows. The next section summarizes the incentive types identified in the literature and how they may affect outcomes. This is followed by a description of the scoping review methodology, results, and conclusion.

## Incentive mechanisms

An incentive is an instrument, payment, or something that motivates to accomplish a task, which may lead to rewards [23]. Four types of incentives have been used to promote the transition towards adopting agroecological practices. They include market, non-market, regulatory, and cross-compliance incentives [2,7]. Market incentives stimulate behavior change by promising economic, environmental, and social benefits through market signals, such as prices, price premiums, input subsidies, and taxes. Non-market incentives are broad in scope, including all incentives that are not market-based, such as technology transfer, technical support, and training. Regulatory incentives promote behavior change through a set of rules, such as environmental laws and mandatory standards imposed by government or private entities to promote agroecology or sustainable production models. Cross-compliance incentives promote behavior change by promising benefits or financial payments to farmers who voluntarily comply with mandatory environmental standards. For example, payment for protecting ecosystem services [2,7].

Incentives can influence outcomes either directly or indirectly through farmers adoption of various agroecological practices [7]. For example, income or profits derived from incentives, such as price premiums, may lead to direct economic and social benefits. Indirectly, incentives may lead to economic (e.g., changes in income), environmental (e.g., reduced carbon emissions), and social (e.g., fair working conditions and increased employment opportunities) outcomes through the adoption of agroecological practices, such as sustainability standards. When farmers receive incentives to promote agroecological practices―as a pathway to creating global sustainable food systems―the indirect pathway is generally expected to play a more robust role. That is, incentives are expected to catalyze the adoption of agroecological practices and principles, ultimately leading to positive economic, environmental, and social outcomes.

## Materials and methods

### Choice of review typology: Narrative, scoping, and systematic review

We conducted a scoping review of the literature to identify the incentives for adopting agroecological practices among rice farmers in Vietnam, and to understand whether and how incentives drive the adoption of agroecological practices and resulting outcomes. The scoping review allowed us to cover all important literature on the topic and minimize the potential bias of a typical narrative review [2]. A narrative review is a scholarly summary of the literature along with interpretations and critiques. Narrative reviews can be conducted using several distinctive methodologies which may vary from the typical methodology of scoping or systematic reviews. Narrative reviews are not *ad hoc* or careless, they can certainly be conducted and presented in a systematic way, depending on purpose, method, and context [24].

A scoping review is a type of knowledge synthesis that follows a systematic approach to map evidence on a topic and identify the main concepts, theories, sources, and knowledge gaps [25]. Scoping reviews offer a unique opportunity to explore aspects such as: (i) the evidence in the literature to address questions relating to what is known about a topic; (ii) what can be synthesized from existing studies to develop policy or practice recommendations; and (iii) what aspects of a topic are yet to be addressed by researchers [2]. A systematic review, as the name implies, is conducted using a systematic, predefined scholarly and critical methodology to identify and analyze the literature on a given topic that falls within the study’s predefined criteria.

Scoping and systematic reviews share several processes, including the use of rigorous and transparent methods to comprehensively identify and analyze all the relevant literature related to a research question [26]. However, they differ in two main ways: (i) a scoping review seeks to present an overview of a potentially large and diverse body of literature related to a broad topic, while a systematic review attempts to collate empirical evidence from a relatively smaller number of studies pertaining to a focused research question [26]; (ii) systematic reviews are useful for answering specific questions with clearly defined hypotheses (for example, “Does this intervention improve specific outcomes when compared with a given comparator in this population?”), whereas scoping reviews are useful for answering questions that are much broader in scope (for example, “What is the nature of the evidence for this intervention?” or “What is known about this concept?”) [25].

Given the difference in objectives, the methodological approaches for scoping and systematic reviews also differ. For instance, systematic reviews require a risk-of-bias assessment for the studies reviewed, whereas this requirement is not mandatory for scoping reviews [25]. Systematic reviews mostly use the Preferred Reporting Items for Systematic reviews and Meta-Analyses (PRISMA) methodology, while scoping reviews use PRISMA with an extension for Scoping Review (PRISMA-ScR) [25,26].

To develop PRISMA-ScR, the original PRISMA statement was adapted, and the following revisions were made: five items were removed (because they were irrelevant to scoping reviews); two items were deemed optional, and the wording was modified for all items [25]. To highlight the differences in the approaches, S1 Table presents the original PRISMA checklist with 27 reporting items, while S2 Table presents the extended PRISMA-ScR checklist with 22 reporting items and modified wordings. Therefore, the PRISMA-ScR approach, which is relevant for scoping reviews was used in this study to answer three broad-scope research questions: “Which incentives are used among rice farmers in Vietnam to promote the adoption of agroecological practices?”, “In what way do the incentives promote the adoption of agroecological practices?” and “How are these incentives related (directly or indirectly) with economic, environmental, or social outcomes among rice farmers in Vietnam?”.

### Search strategy and inclusion criteria

To conduct this review, we followed the PRISMA-ScR guidelines/protocol, which provides detailed guidelines on how to assess the scope of literature on a topic and synthesize evidence when conducting a review [27].

Our scoping review was conducted systematically, using keyword searches in three literature databases—Cab Abstracts, Google Scholar, and Scopus—to identify the literature for inclusion in this review. The keywords searched (individually and in combination) were: incentives; agroecology; agroecological; biodiversity conservation; climate change; climate smart agriculture; conservation agriculture; rice; sustainable agriculture; and Vietnam. The exact search-term combinations are presented in S3 Table. We screened the titles of the articles returned by the keyword search terms. This was then followed by screening of the abstracts and full texts of the articles returned. We also screened the reference lists of eligible articles to reduce the chances of missing potentially relevant studies. Title, abstract, and full-text screening was conducted by two independent reviewers to reduce the risk of bias and ensure that only relevant articles that fulfilled the inclusion/exclusion criteria (specified below) were included in the review. When there was doubt regarding whether to include or exclude an article, the two reviewers discussed the articles and screened them against the set of inclusion criteria before reaching a consensus on which to keep or reject.

We applied six eligibility criteria for this review: (i) articles published between January 2012 and December 2022; (ii) articles that explicitly focused on incentives for agroecological practices among rice farmers in Vietnam; (iii) articles that explicitly focused on the adoption of agroecological practices among rice farmers in Vietnam; (iv) articles that explicitly associated the adoption of agroecological practices with economic, environmental, or social outcomes among rice farmers in Vietnam; (v) articles that explicitly analyzed the effects of incentives on outcomes among rice farmers in Vietnam; and (vi) English-language original research, peer-reviewed articles, dissertations, and theses, including grey literature (from reputable organizations) on incentives and agroecology.

### Data extraction, management, and analysis

After completing the article screening and selection process, relevant data on the authors’ names, publication title, and year of publication were recorded to avoid duplication. We then extracted and summarized details of the incentive type, agroecological practices, and outcomes—the effects or consequences of the incentives or adoption of agroecological practices were classified into either economic, environmental, or social domains—from each article and recorded them in a Microsoft Excel spreadsheet for analysis. The two independent reviewers began the coding with a set of pre-established codes (deductive coding) for some variables, such as type of incentive used and the type of outcomes likely to be affected by incentives. The two reviewers also created a set of codes based on emerging insights from the review data itself (inductive coding). This was done for variables, such as the specific types of incentives used, the agroecological practices adopted, and the specific outcomes achieved as a result of adopting agroecological practices. The two reviewers also rapidly reviewed the articles and the coded or summarized data a second time to verify and triangulate the results.

Deductive coding allows approaching the analysis with a focused lens and to quickly identify relevant data, but its downside is that valuable insights could be overlooked due to its tight, predetermined focus [28]. Deductive coding is relevant where literature on a topic is available. Inductive coding is more relevant where the literature is not well developed, and researchers want to investigate new ideas and concepts. Inductive coding allows developing themes based on what is emerging from the data. Its drawback is that it may be time-consuming, as it is not narrowly focused and allows the researcher to go with the data flow. Therefore, to approach this review with a focused lens, and to quickly identify relevant data, while also investigating new ideas and concepts, we used a hybrid coding approach, combining both deductive and inductive coding or a summary of the data [28].

After data coding, we examined the direct linkages between incentives and outcomes. To understand how incentives motivate the adoption of agroecological practices, and how adopting specific practices leads to the desired outcomes, we conducted an in-depth review of articles that provided a clear association between incentives, adoption, and outcomes (details below). The data summarized and extracted in Microsoft Excel (S1 File) were used to draw the graphs (Fig 2 and Fig 3) and Sankey diagrams (Fig 4 and Fig 5) presented in the results section of this paper. A Sankey diagram is a data visualization technique used to depict flow, movement, or change from one set of values to another. Sankey diagrams have recently been used to examine complex multi-step processes.

## Results and discussion

The results of this study, presented below, fully address our objective of reviewing the literature and understanding the incentives used to promote the adoption of agroecological practices among rice farmers in Vietnam, whether and in what way the incentives promote the adoption of agroecological practices, and the resulting outcomes of these practices.

### Article screening

**Fig 1:**
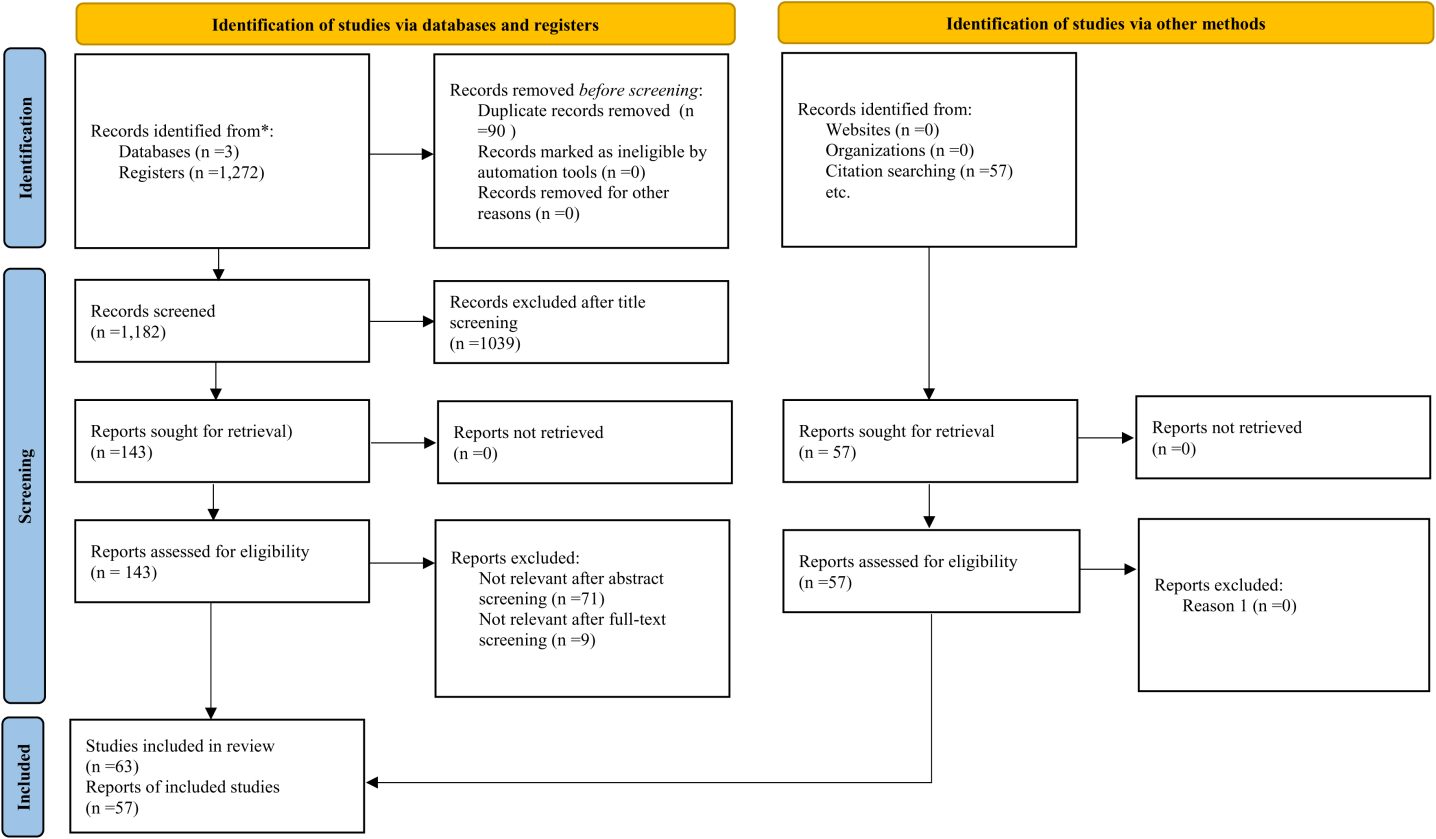
Scoping review flow diagram. Adapted from Page et al. (2021).

The keyword search terms used during article screening following the PRISMA-ScR protocol returned 1,272 articles—991 articles from Google Scholar, 169 from Cab Abstracts, and 112 from Scopus. After removing 90 duplicate articles, 1,182 were available for title, abstract, and full-text screening. Title screening resulted in a first selection of 143 articles meeting the inclusion criteria; this was reduced to 72 articles retained after abstract screening, and then 63 articles being retained after the full-text screening. Finally, to reduce the likelihood of excluding relevant studies, we screened the references lists of the 143 eligible articles, from which we selected 57 articles, adding them to the 63 selected, resulting in a total of 120 articles for full review. The number of articles at each screening stage is captured in Fig 1.

**Fig 2:**
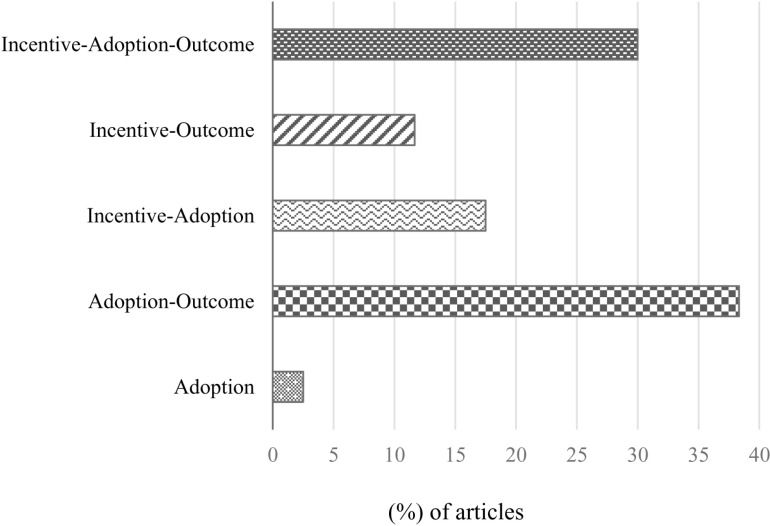
Share (%) of selected articles and the topics or linkages they cover.

The 120 articles retained from these screenings were assessed for the positive association between incentives and the adoption of agricultural practices, and/or outcomes. This assessment enabled a deeper review of the incentive types, the agroecological practices adopted, and the outcomes among rice farmers in Vietnam. The share (%) of selected articles and the topics they cover are presented in Fig 2.

The results of our assessment show that the largest share (38%) of the 120 articles retained for the study focused on the association between adopting agroecological practices and outcomes. About 30% of the articles associated incentives with adopting agroecological practices and outcomes, which suggests a sizeable gap in the literature on studies that explicitly associate incentives with the adoption of agroecological practices and outcomes. About 18% of the articles correlated incentives with adoption, 12% correlated incentives with outcomes, while 2% of the reviewed articles correlated incentives only with adopting agroecological practices. Approximately 60% of the articles correlated incentives with adoption, outcomes, or both, suggesting a common use of incentives, and a research interest in the incentives among rice farmers in Vietnam.

### Adopted practices

**Fig 3:**
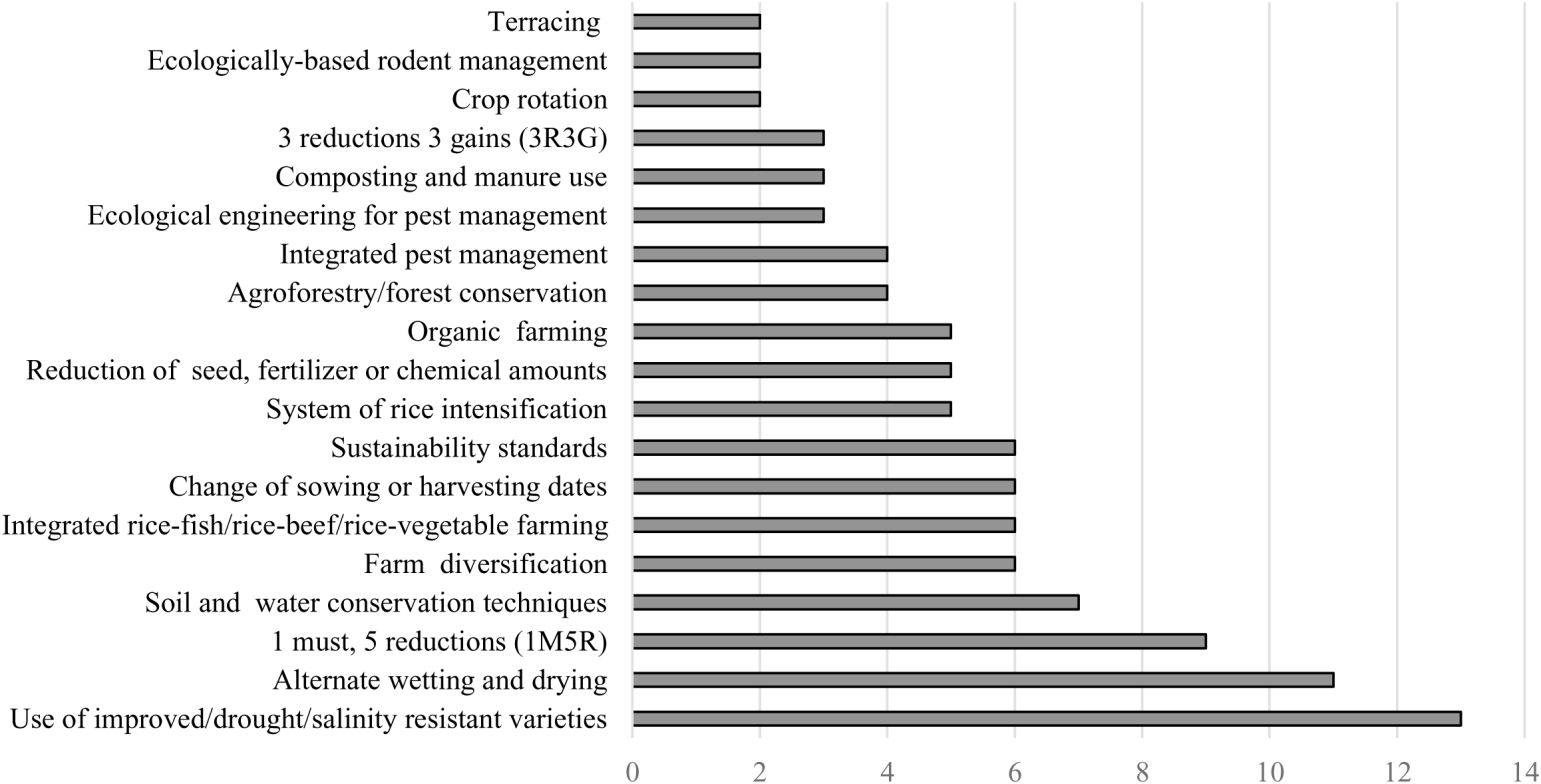
Summary of the number of agroecological practices covered by articles included in the review.

Fig 3 shows the agroecological farming practices implemented by Vietnamese rice farmers in the 120 relevant articles focused on rice production. The practices include using drought- and salinity-tolerant varieties, AWD, 1M5R, soil and water conservation techniques, farm diversification, rice−fish/rice−beef/rice−vegetable farming, changing sowing or harvesting dates, sustainability standards, a system of rice intensification, reducing seed, fertilizer, or chemicals used, organic rice farming, agroforestry/forest conservation, integrated pest management, ecological engineering for pest management, composting and manure application, 3R3G, crop rotation, ecologically-based rodent management, and terracing. Results show that using drought- and salinity-tolerant varieties (13), AWD (11), and 1M5R (9) were the most frequently implemented practices, while crop rotation (2), ecologically-based rodent management (2), terracing (2) were the least implemented practices in the reviewed articles. It is plausible that the most frequently implemented practices—drought- and salinity-tolerant rice varieties, AWD, and 1M5R—are due to substantial efforts to promote them [13,14].

### Determinants of agroecological practice adoption

A review of whether and how incentives motivate the adoption of agroecological practices revealed that incentives are crucial for promoting the adoption of agroecological practices, as farmers were reluctant to adopt practices without them [14,29,30]. Fifty-seven articles in the review showed that incentives were used among rice farmers in Vietnam to encourage the adoption of agroecological practices, such as the AWD, 1M5R, 3R3G and organic rice farming [14,30]. For instance, membership in paddy cooperatives, information provision or awareness campaigns, and training were successfully used (positively associated with increased adoption) to promote adoption of AWD and 1M5R [14]. Extension services, training, and information provision were also used and found to be positively associated with the adoption of integrated pest management, row seeding, 3R3G, certified seeds, and new rice varieties [10,11,30]. Technical training and awareness creation of organic farming were associated with increased adoption of organic rice farming [30]. Providing extension services or training increases farmers’ understanding of the agroecological practices and instills confidence in them to sufficiently adopt the practices. Land-tenure security was associated with increased probability of adopting agroecological practices [10,11]. This finding is consistent with the results of Maguza-Tembo et al. [31], who found that tenants were less likely to apply new technologies on rented plots in the absence of tenure security. Land tenure security guarantees the right to access and use of land, which can promote investment in land, including adopting agroecological practices.

Farmers’ adoption decisions are also a function of their experiences with agroecological practices, such that present farm-level practices influence their willingness to adopt newer ones. Farmers’ characteristics, especially risk attitudes and cultural preferences, education, farm sizes, economic status, and environmental consciousness (e.g., knowledge of their land and soil quality) affect their adoption decisions [10–12]. For instance, this review found that farmers who experienced problems with their land or had poor soil quality were more likely to adopt agroecological practices, such as soil and water conservation practices, organic farming, crop rotation, and land fallowing [10,11]. Labor and financial constraints also affect the adoption of agroecological practices. For instance, the availability of labor significantly encouraged the adoption of organic fertilizers and soil and water conservation practices, which are labor-intensive practices [11].

Farmers are keen to understand the agroecological practices and their effectiveness or benefits before adopting them. At least 12 articles in this review found that increasing farmers awareness and understanding of the practices (and their benefits) by providing technical assistance and sharing information (e.g., training and knowledge transfer through agricultural extension agents and peer learning) enhanced the adoption of agroecological practices [10,11,13,14,30,32–38]. Pham et al. [11] found that social learning through informal groups, such as farmer groups and sharing information with neighbors and friends increased the likelihood of adopting crop rotation and organic farming. The combination of multiple social learning channels further increased the probability of adopting crop rotation and organic farming by 6 and 21 percentage points, respectively, underlining the vital role of social learning in encouraging the diffusion of agricultural technologies in Vietnam. These findings suggest that cooperation among extension agents, farmer groups, and peers is needed to encourage adoption of agroecological practices. Membership in farmer groups can enhance peer learning and encourage adoption of agroecological practices. Although training as an incentive was important, the know-how or quality of extension agents or training was a more important driver of adoption than simply offering training [10]. This finding is consistent with that of Teklewold et al. [39], who found that extension contact alone did not increase adoption but rather the quality of the extension workers did. Successful extension requires well-developed programs and capable professionals to train farmers.

### Linkages among incentives, adoption, and outcomes

**Fig 4:**
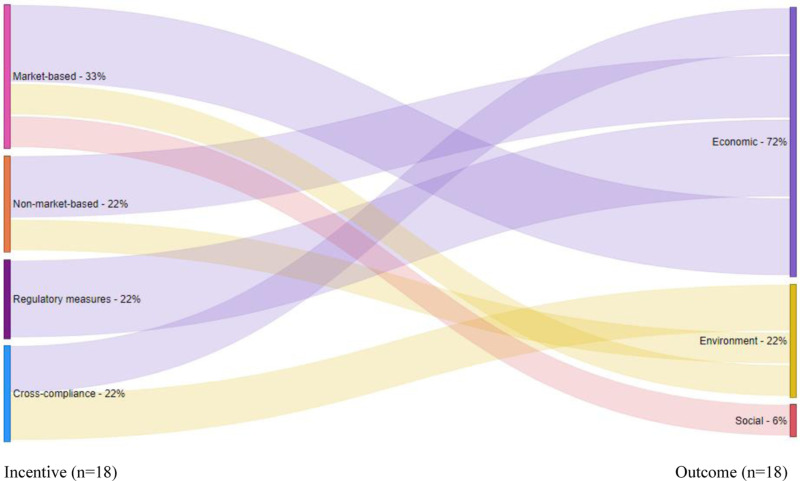
The relationship between incentives and outcomes (incentive‒outcome linkages).

Here we present the results of the linkages among incentives, adoption, and outcomes. Incentives can influence outcomes indirectly by serving as a catalyst for the adoption of agroecological principles and practices, ultimately leading to outcomes. Incentives can also affect outcomes directly. Fewer articles in this review (14) focused on direct linkages between incentives and outcomes compared to those with indirect linkages between incentives and outcomes (36). Fig 4 shows the direct relationship between multiple types of incentives and outcomes. About 33% of the incentives with direct outcome linkages were market-based. 22% were non-market, while 22% were regulatory, and 22% were cross-compliance incentives. Market incentives are the most-frequently used incentives among incentives with direct outcome linkages, while non-market, regulatory and cross-compliance incentives are relatively less common among rice farmers in Vietnam. The interest in market incentives may be due to their flexibility and tendency to contribute to long-term solutions [40]. Examples of the most frequently used market incentives among rice farmers in Vietnam include input subsidies, credit for inputs, price premiums, and contract farming. These incentives have been associated with some direct benefits or outcomes. For instance, Luan and Bauer [18] found that access to credit increased farmers’ household income. Duy [41] found a positive association between credit and rice production efficiency. Tuyen et al. [42] found that farmers prefer farming contracts with price premiums for sustainably produced rice because they contribute to increased incomes. Le Ngoc [19] found that contract farming was associated with increased technical efficiency among rice farmers.

Like market incentives, non-market incentives are generally preferred in the literature because they tend to be broad in scope and flexible [2]. Fig 4 shows that 22% of the incentives with direct linkages to outcomes were non-market incentives. Common non-market incentives used to promote agroecological principles include training, extension, and collective action through membership in producer organizations. These incentives facilitate access to information and credit. They can also subsidize the cost of inputs by reducing transaction costs. Direct benefits of the non-market incentives were also observed among rice farmers in Vietnam. For instance, Rejesus et al. [43] found that access to extension through farmer field schools increased farmers’ awareness and knowledge of integrated pest management practices. Tran et al. [21] found that membership in farmer cooperatives increased rice farmers’ returns.

Fig 4 reveals that 22% of the incentives with direct outcome linkages were regulatory incentives. Regulatory incentives often promote behavioral change through compulsory rules or regulations (e.g., environmental laws and standards). They are sometimes perceived as too complex and inflexible compared to short-term policy instruments that provide financial support, often making them less popular than other incentives [44]. Due to their perceived complexity, they are often accompanied by technical assistance and training to increase their effectiveness [2]. Examples of regulatory incentives used among rice farmers in Vietnam to promote agroecological principles include certification (e.g., certification for organic rice farming), land tenure or land use rights, and sustainability standards [16,32,45,46]. They also include participatory guarantee systems, which are alternatives to third-party certification schemes with lower entry costs [47]. These incentives also directly influenced outcomes. For instance, Nguyen [45] found that land tenure rights improved access to agricultural credit, while Sato et al. [46] found that certification in good agricultural practices (GAP standards) was associated with higher net returns and reduced environmental footprints.

Like non-market and regulatory incentives, 22% of the incentives with a direct link to outcomes are cross-compliance incentives (Fig 4). Cross-compliance incentives encourage adoption by providing payments for ecosystem services (PES) or rewards to farmers who voluntarily comply with basic environmental standards. Most PES are state-run and are found in developed countries [48], which potentially explains why cross-compliance incentives have received relatively less frequent attention among rice farmers in Vietnam. Regarding the direct impacts on outcomes, the PES used among rice farmers in Vietnam have reported mixed results. For instance, Tuijnman et al. [49] found that PES contribute to environmental protection but are also associated with declining incomes and livelihood opportunities for women, especially those relying on timber and non-timber forest products. Thuy and Duong [50] also found that while some surveyed households have experienced income gains from PES, incomes for other households had declined. Looking at the four types of incentives— market, non-market, regulatory, and cross-compliance—the results suggest that market incentives were the most important for directly influencing outcomes.

Fig 4 also shows that the incentives were most associated with economic outcomes (72%), such as changes in profit and income, than with environmental outcomes (22%), such as reduced deforestation or carbon footprints. The incentives were least associated with social outcomes (6%). The analysis of how each incentive type was directly associated with the outcomes showed that market and non-market incentives were more associated with economic outcomes, followed by environmental and social outcomes. Non-market incentives were not associated with social outcomes. Regulatory incentives were exclusively linked to economic outcomes. Cross-compliance incentives were evenly associated with economic and environmental outcomes but not with social outcomes. These results suggest that incentives influence outcomes differently, and a combination of different incentive types is required to achieve holistic outcomes.

**Fig 5:**
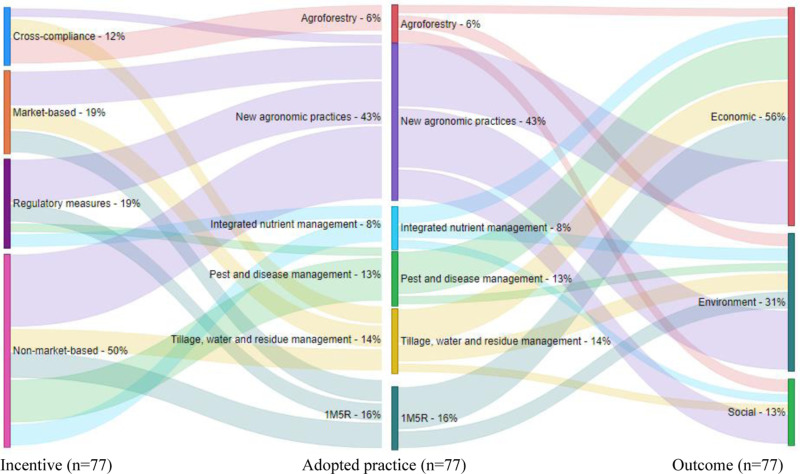
The relationship between incentives, adoption of practices, and outcomes (incentive‒adoption‒outcome linkages).

Fig 5 presents the results of the indirect relationship between incentives and outcomes through the adoption of agroecological practices among rice farmers in Vietnam. Contrary to the research and development literature on the direct incentive-outcome linkages (Fig 4), non-market incentives were the most prevalent (50%) incentives studied with indirect-outcome linkages. Market and regulatory incentives were assessed at about the same (19%) frequency, while cross-compliance incentives were the least-frequently studied incentives (12%) to drive practice adoption and ultimately outcomes. These results suggest that non-market incentives were in general the most important type of incentives for influencing adoption of agroecological practices and outcomes among rice farmers in Vietnam. Cross-compliance incentives were the least frequently assessed, consistent with the literature which shows that compliance incentives are less common in developed countries [48].

Regarding the agroecological practices adopted (Fig 3), we grouped them into six categories following a modified Intergovernmental Panel on Climate Change (IPCC) [51] categorization. We then linked them to the incentive categories. The six agroecological practice categories were (i) agroforestry (6%), (ii) integrated nutrient management (8%), (iii) pest and disease management (13%), (iv) tillage, residue, and water management (14%), (v) 1M5R (16%), and (vi) new agronomic practices (43%). Consistent with the findings of Mockshell et al. [7], the results show that market, non-market, and regulatory incentives were more associated with new agronomic practices, such as farm diversification, improved varieties or technologies, crop rotation, and organic agriculture. Cross-compliance incentives were more associated with agroforestry. This is plausible as cross compliance incentives, especially PES, are mostly used to promote environmental outcomes. Agroforestry was more associated with promotion of environmental and social outcomes than with economic outcomes. Some of the environmental benefits of agroforestry include mitigation of soil erosion and improvement of soil moisture and fertility [52]. 1M5R practices were more associated with economic than environmental outcomes, but not with social outcomes. For instance, 1M5R practices improved farmers economic outcomes by reducing their production cost by 10%, increasing their paddy’s selling price by 4.5% per kg, and increasing their profit by 10%, compared to traditional farming households [13]. Integrated nutrient management and new agronomic practices contributed more to economic and environmental than social outcomes. Tillage and water management contributed more to economic outcomes than environmental and social outcomes, while pest and disease management practices were more associated with economic outcomes. In terms of the link between incentives and outcomes, the results show that, in general, the incentives were more indirectly associated with economic outcomes (56%) than with environmental (31%) and social (13%) outcomes. The results show that studies linking incentives to agroecological practices and outcomes mostly focus on examining economic outcomes, with social outcomes receiving the least attention. These results underline that different types of incentives affect adoption and outcomes differently. A combination of incentives is therefore required to achieve holistic outcomes. Incentives that promote social outcomes are especially needed.

## Conclusion and policy implications

This scoping review reveals that incentives play an important role in promoting the adoption of agroecological practices. Market, non-market, regulatory, and cross compliance incentives were the main categories of incentives used to promote the adoption of agroecological practices. Market incentives were more associated with the outcomes directly, while non-market incentives were more important for influencing outcomes indirectly through the adoption of agroecological practices among rice farmers in Vietnam. Farmer organizations, access to agricultural credit, contracts guaranteeing premium prices, technical assistance, extension services or training, land-tenure security, awareness campaigns and peer learning emerged as important specific incentives for promoting the adoption of agroecological practices and outcomes. These incentives mainly enhanced the adoption of agroecological practices by facilitating peer learning and knowledge sharing about agroecological practices, relaxing financial constraints to adoption of practices, signaling higher returns on adopted practices through premium prices, increasing farmers awareness and understanding of the benefits of practices, ensuring land-tenure security that can enhance investments in land. The review also finds that there is greater focus on economic and environmental outcomes than on social outcomes.

Overall, this scoping review shows that incentives are important for promoting the adoption of agroecological practices and catalyzing the transition toward sustainable production systems. This implies that incentives should be incorporated in all activities that aim to promote the uptake of agroecological practices. Our findings also suggest that different types of incentives are important for promoting different agroecological practices. Therefore, policymakers, development practitioners, and researchers should use a combination of incentive types to achieve holistic outcomes and food system transformation. Moreover, interventions designed to promote the adoption of agroecological practices among farmers should be accompanied with relevant or proven incentives to expedite uptake. For instance, the review revealed that membership in paddy cooperatives, information provision or awareness campaigns, technical assistance and training or extension have been used successfully to promote the adoption of agroecological practices, such as AWD, 1M5R, integrated pest management, row seeding, 3R3G, crop rotation, organic farming and improved rice varieties. This implies that such incentives could be important when promoting similar practices in the future. Training and technical assistance were found to be important incentives when implementing regulatory incentives perceived as complex, such as certification, land use rights, and sustainability standards. Our findings highlight the need for investment in quality training and extension services to increase farmers’ awareness and understanding of agroecological practices and their benefits.

Since farmers often aim to maximize certain benefits or outcomes, policymakers, development practitioners, and researchers should consider using incentive mechanisms with fewer requirements or costs to increase the adoption of agroecological practices and their benefits. Incentives should promote all outcomes, especially social outcomes which have received less attention in the literature. Future studies should examine the relationships between cross-compliance incentives, adoption, and social outcomes, which were less studied. Apart from the specific incentives revealed in this review, our analysis points to the importance of improving other factors―such as agricultural institutions, policies and regulations, infrastructure and markets, off-farm employment opportunities, structural poverty and the scarcity of asset endowments―as they influence farmers’ capacity and willingness to invest in sustainable agricultural practices.

## Supporting information

S1 Table PRISMA 2020 checklist for systematic reviews. (DOCX)

S2 Table Preferred reporting items for systematic reviews and meta-analyses extension for scoping reviews (PRISMA-ScR) checklist (the 5 items dropped from the PRISMA checklist are highlighted in yellow). (DOCX)

S3 Table Database search strategy. (DOCX)

S4 Table List of articles that underwent abstract and full text screening. (DOCX)

S1 File Data file. (XLSX)
