# Supplementary material for: A scoping review of the incentives for promoting the adoption of agroecological practices and outcomes among rice farmers in Vietnam
Source: PLoS One. 2025 Apr 25;20(4):e0321029. doi: 10.1371/journal.pone.0321029 (PMC12027032; doi:10.1371/journal.pone.0321029)
Supplement: S3 Table — (DOCX) [file pone.0321029.s003.docx]

**S3 Table. Database search strategy**

| Database | Search terms | Search results |
| --- | --- | --- |
| Google Scholar | Incentives ("Agroecology" OR "Agroecological") "Sustainable Agriculture" OR "Conservation Agriculture" OR "Biodiversity Conservation" OR "Climate Change" OR "Climate-Smart Agriculture” Vietnam AND Rice  (PUBYEAR > 2012 and PUBYEAR < 2022) | 991 |
| CAB Abstracts | (ab:("Agroecology" OR "Agroecological" OR "Sustainable Agriculture" OR "Conservation Agriculture" OR "Biodiversity Conservation" OR "Climate Change" OR "Climate-smart Agriculture" OR "Incentives") AND ab:("vietnam") AND ab:("rice") AND yr:[2012 TO 2022]) AND ( ((language:(( "English" ) )) (geographic-location:(( "Vietnam" ) )) )) | 169 |
| SCOPUS | (TITLE-ABS-KEY ("Agroecology" OR "Agroecological" OR "Sustainable Agriculture" OR "Conservation Agriculture" OR "Biodiversity Conservation" OR "Climate Change" OR "Climate-Smart Agriculture" OR "Incentive*") AND TITLE-ABS-KEY ("Rice") AND TITLE-ABS-KEY ("Vietnam")) AND PUBYEAR > 2012 AND PUBYEAR < 2022 AND PUBYEAR > 2012 AND PUBYEAR < 2022) | 112 |
