# Supplementary material for: A scoping review of the incentives for promoting the adoption of agroecological practices and outcomes among rice farmers in Vietnam
Source: PLoS One. 2025 Apr 25;20(4):e0321029. doi: 10.1371/journal.pone.0321029 (PMC12027032; doi:10.1371/journal.pone.0321029)
Supplement: S4 Table — (DOCX) [file pone.0321029.s004.docx]

**S4 Table. List of articles that underwent abstract and full text screening.**

| 1. Thakur, A. K., & Uphoff, N. T. (2017). How the system of rice intensification can contribute to climate‐smart agriculture. Agronomy Journal, 109(4), 1163-1182. |
| --- |
| 1. Horgan, F. G., Vu, Q., Mundaca, E. A., & Crisol-Martínez, E. (2022). Restoration of rice ecosystem services:‘ecological engineering for pest management’incentives and practices in the Mekong Delta Region of Vietnam. *Agronomy*, *12*(5), 1042. |
| 1. Uphoff, N. An Agroecological Route to Agricultural Development. *Linking Science to Society*, 3255. |
| 1. Da, C. T., Phuoc, L. H., Duc, H. N., Troell, M., & Berg, H. (2015). Use of wastewater from striped catfish (Pangasianodon hypophthalmus) pond culture for integrated rice–fish–vegetable farming systems in the Mekong Delta, Vietnam. *Agroecology and Sustainable Food Systems*, *39*(5), 580-597. |
| 1. Mishra, A., Ketelaar, J. W., Uphoff, N., & Whitten, M. (2021). Food security and climate-smart agriculture in the lower Mekong basin of Southeast Asia: Evaluating impacts of system of rice intensification with special reference to rainfed agriculture. *International Journal of Agricultural Sustainability*, *19*(2), 152-174. |
| 1. Tu, V. H., Can, N. D., Takahashi, Y., Kopp, S. W., & Yabe, M. (2018). Modelling the factors affecting the adoption of eco-friendly rice production in the Vietnamese Mekong Delta. *Cogent Food & Agriculture*, *4*(1), 1432538. |
| 1. Tivet, F., & Boulakia, S. (2017). Climate Smart Rice Cropping systems in Vietnam. State of knowledge and prospects. |
| 1. Lovell, R. J., Shennan, C., & Thuy, N. N. (2021). Sustainable and conventional intensification: how gendered livelihoods influence farming practice adoption in the Vietnamese Mekong River Delta. *Environment, Development and Sustainability*, *23*, 7089-7116. |
| 1. Farnworth, C. R., Hà, T. T., Sander, B. O., Wollenberg, E., De Haan, N. C., & McGuire, S. (2017). Incorporating gender into low-emission development: a case study from Vietnam. *Gender, Technology and Development*, *21*(1-2), 5-30. |
| 1. Nguyen, H. Q. (2017). Analyzing the economies of crop diversification in rural Vietnam using an input distance function. *Agricultural systems*, *153*, 148-156. |
| 1. Hoang, V. N., Nguyen, T. T., Wilson, C., Ho, T. Q., & Khanal, U. (2021). Scale and scope economies in small household rice farming in Vietnam. *Journal of Integrative Agriculture*, *20*(12), 3339-3351. |
| 1. Thi, Q. A. L., Shimamura, Y., & Yamada, H. (2021). Information acquisition and conservation farming practices for sustainable agriculture in rural Vietnam. *Asian Journal of Agriculture and Development*, *18*(1), 32-48. |
| 1. Andrea, P. (2018). Potentials of system of rice intensification (SRI) in climate change adaptation and mitigation. A review. |
| 1. Bui, H. T. M., & Do, T. A. (2022). Choice of adaptation strategies to climate change among farm households in mountainous areas of Northeastern Vietnam. *GeoJournal*, *87*(6), 4947-4960. |
| 1. Hoang, M., Van Noordwijk, M., Fox, J., Thomas, D., Sinclair, F., Catacutan, D., ... & Simons, T. (2014). Are trees buffering ecosystems and livelihoods in agricultural landscapes of the Lower Mekong Basin. *Consequences for Climate-Change Adaptation. World Agroforestry Centre (ICRAF) Southeast Asia Regional Program, Bogor, Indonesia*. |
| 1. Khai, H. V., Utsunomiya, Y., Khong, T. D., & Khoi, L. N. D. (2022). Do Neighbors Affect People's Demand for the Biodiversity Conservation Project in the U Minh Ha Peat Swamp Forest of the Mekong Delta, Vietnam?. *Frontiers in Sustainable Food Systems*, *5*, 808117. |
| 1. Lan, N. T. P., & Van Kien, N. (2021). Back to nature-based agriculture: green livelihoods are taking root in the Mekong River Delta. *Journal of People, Plants, and Environment*, *24*(6), 551-561. |
| 1. Choi, J., Uphoff, N., Kim, J., & Lee, S. (2019). Greenhouse Gas Reduction from Paddy by Environmentally-Friendly Intermittent Irrigation: A Review. *Journal of Wetlands Research*, *21*(1), 43-56. |
| 1. Cassou, E. (2018). The Greening of Farm Support Programs: International Experience s with Agricultural Subsidy Reform. *World Bank, Washington, DC*. |
| 1. Böttinger, C. (2019). Self-Organized Community Institutions: A Way to Strengthen Local Social Sustainability? A Case Study of PGS Viêt Nam. |
| 1. Chi, T. T. N. (2008). Factors affecting technology adoption among rice farmers in the Mekong Delta through the lens of the local authorial managers: an analysis of qualitative data. *Omonrice*, *16*, 107-112. |
| 1. Atieno, M., Herrmann, L., Nguyen, H. T., Phan, H. T., Nguyen, N. K., Srean, P., ... & Lesueur, D. (2020). Assessment of biofertilizer use for sustainable agriculture in the Great Mekong Region. *Journal of environmental management*, *275*, 111300. |
| 1. Pham, H. G., Chuah, S. H., & Feeny, S. (2021). Factors affecting the adoption of sustainable agricultural practices: Findings from panel data for Vietnam. Ecological Economics, 184, 107000. |
| 1. Umetsu, C. (2021). “One must do, five reductions” technical practice and the economic performance of rice smallholders in the Vietnamese Mekong delta. *Sustainable Production and Consumption*, *28*, 1040-1049. |
| 1. Flor, R. J., Tuan, L. A., Hung, N. V., My Phung, N. T., Connor, M., Stuart, A. M., ... & Singleton, G. R. (2021). Unpacking the processes that catalyzed the adoption of best management practices for lowland irrigated rice in the Mekong Delta. *Agronomy*, *11*(9), 1707. |
| 1. Luu, D. T. (2020). Origins of farmers’ adoption of multiple climate-smart agriculture management practices in the Vietnamese Mekong Delta. *Makara Human Behavior Studies in Asia*, *24*(2), 141-153. |
| 1. Hoang, H. G. (2021). Determinants of adoption of organic rice production: a case of smallholder farmers in Hai Lang district of Vietnam. *International Journal of Social Economics*, *48*(10), 1463-1475. |
| 1. Nguyễn, N. T., & Hoang, H. A. (2022). Factors influencing the adoption of “One must do, five reductions” in rice production in the Mekong River Delta: A case study in Soc Trang province, Vietnam. *Tạp chí Nông nghiệp và Phát triển*, *21*(3), 12-20. |
| 1. Castella, J. C., & Kibler, J. F. (2015). *Towards an agroecological transition in Southeast Asia: Cultivating diversity and developing synergies*. GRET. |
| 1. Stuart, A. M., Devkota, K. P., Sato, T., Pame, A. R. P., Balingbing, C., Phung, N. T. M., ... & Singleton, G. R. (2018). On-farm assessment of different rice crop management practices in the Mekong Delta, Vietnam, using sustainability performance indicators. *Field Crops Research*, *229*, 103-114. |
| 1. Tu, V. H., Can, N. D., Takahashi, Y., Kopp, S. W., & Yabe, M. (2019). Technical and environmental efficiency of eco-friendly rice production in the upstream region of the Vietnamese Mekong delta. *Environment, Development and Sustainability*, *21*, 2401-2424. |
| 1. 山口哲由, 南川和則, & 横山繁樹. (2016). Alternate wetting and drying (AWD) irrigation technology uptake in rice paddies of the Mekong Delta, Vietnam: relationship between local conditions and the practiced technology. *アジア・アフリカ地域研究*, *15*(2), 234-256. |
| 1. Tuan, L. A., Wehmeyer, H., & Connor, M. (2022). “One must do, five reductions” qualitative analysis of the diffusion and adoption constraints in Vietnam. *Development in Practice*, *32*(6), 768-780. |
| 1. Phi, H. D., Dinh, P. H., & Quang, M. B. (2021). Factors influencing new technology adoption behaviors of rice farmers: Binary logistic regression model approach. *International Journal of Business and Management Review*, *9*(4), 54-71. |
| 1. Connor, M., Cuong, O. Q., Demont, M., Sander, B. O., & Nelson, K. (2022). The influence of climate change knowledge on consumer valuation of sustainably produced rice in Vietnam. *Sustainable Production and Consumption*, *31*, 1-12. |
| 1. Umetsu, C. (2022). Rice variety and sustainable farming: A case study in the Mekong Delta, Vietnam. *Environmental Challenges*, *8*, 100532. |
| 1. Lan, L., Sain, G., Czaplicki, S., Guerten, N., Shikuku, K. M., Grosjean, G., & Läderach, P. (2018). Farm-level and community aggregate economic impacts of adopting climate smart agricultural practices in three mega environments. *Plos one*, *13*(11), e0207700. |
| 1. Bosma, R. H., Nhan, D. K., Udo, H. M., & Kaymak, U. (2012). Factors affecting farmers’ adoption of integrated rice–fish farming systems in the Mekong delta, Vietnam. *Reviews in Aquaculture*, *4*(3), 178-190. |
| 1. Dung, L. T., Phi Ho, D., Thi Kim Hiep, N., & Hoi, P. T. (2018). The Determinants of Rice Farmers Adoption of Sustainable Agricultural Technologies in the Mekong Delta, Vietnam. Applied Economics Journal, 25(2), 55-69. |
| 1. Tran, N. Q., Ngo, T. V., Nguyen, N. V., Duong, T. N., Nguyen, C. D., Quach, T. D., & Le, D. V. (2022). Impact of New-Type Agricultural Cooperatives on Profitability of Rice Farms: Evidence from Vietnam’s Mekong River Delta. Economies, 10(12), 306. |
| 1. Dinh, N. C., Mizunoya, T., Ha, V. H., Hung, P. X., Tan, N. Q., & An, L. T. (2023). Factors influencing farmer intentions to scale up organic rice farming: preliminary findings from the context of agricultural production in Central Vietnam. *Asia-Pacific Journal of Regional Science*, 1-26. |
| 1. Van Song, N., Cuong, H. N., Huyen, V. N., & Rañola Jr, R. F. (2020). The determinants of sustainable land management adoption under risks in upland area of Vietnam. *Sustainable Futures*, *2*, 100015. |
| 1. Tuan, C. M., & Lee, S. H. (2021). Factors Affecting Organic Fertilizer Adoption in Rice Production in Vietnam. *한국국제농업개발학회지*, *33*(2), 130-138. |
| 1. Kien, N. D., Dung, T. Q., Oanh, D. T. K., An, L. T., Dinh, N. C., Phan, N. T., & Nga, L. T. T. (2023). Climate‐resilient practices and welfare impacts on rice‐cultivating households in Vietnam: Does joint adoption of multiple practices matter?. Australian Journal of Agricultural and Resource Economics, 67(2), 263-284. |
| 1. Ferrer, A. J. G., Thanh, L. H., Chuong, P. H., Kiet, N. T., Trang, V. T., Duc, T. C., ... & Bernardo, E. B. (2023). Farming household adoption of climate-smart agricultural technologies: evidence from North-Central Vietnam. *Asia-Pacific Journal of Regional Science*, 1-23. |
| 1. Nam, L. P., Dang Que, N., Van Song, N., Hoang Mai, T. T., Minh Phuong, N. T., Xuan Huong, N. T., ... & Uan, T. B. (2022). Rice farmers' perception and determinants of climate change adaptation measures: a case study in Vietnam. |
| 1. Duc, K. N., Ancev, T., & Randall, A. (2021). Farmers' choices of climate-resilient strategies: Evidence from Vietnam. *Journal of Cleaner Production*, *317*, 128399. |
| 1. Huan, N. H., Thiet, L. V., Chien, H. V., & Heong, K. L. (2005). Farmers’ participatory evaluation of reducing pesticides, fertilizers and seed rates in rice farming in the Mekong Delta, Vietnam. *Crop Protection*, *24*(5), 457-464. |
| 1. Bauer, S. (2016). Does credit access affect household income homogeneously across different groups of credit recipients? Evidence from rural Vietnam. *Journal of rural studies*, *47*, 186-203. |
| 1. Newman, C., Tarp, F., & Van Den Broeck, K. (2015). Property rights and productivity: The case of joint land titling in Vietnam. *Land Economics*, *91*(1), 91-105. |
| 1. Ha, T. M., & Bac, H. V. (2021). Effects of climate-smart agriculture adoption on performance of rice farmers in Northeast Vietnam. *Asian Journal of Agriculture and Rural Development*, *11*(4), 291-301. |
| 1. Nguyen Chau, T., & Scrimgeour, F. (2022). Productivity impacts of hybrid rice seeds in Vietnam. *Journal of Agricultural Economics*, *73*(2), 414-429. |
| 1. Thanh, P. T., & Duong, P. B. (2021). Economic impacts of hybrid rice varieties in vietnam: An instrumental analysis. *Journal of Agricultural Science and Technology*, *23*(6), 1195-1211. |
| 1. Tran, H. L. (2020). *Farmer participation and irrigation performance: A case study of Nam Thach Han irrigation system, Vietnam* (Doctoral dissertation, The University of Waikato). |
| 1. Le Anh, T. (2009). *Socio-economic constraints to rice farmers’ adoption of the community trap barrier system for controlling rodents in rice-based farming systems in the Mekong Delta, Vietnam* (Doctoral dissertation, James Cook University). |
| 1. Palis, F., Sumalde, Z., Torres, C., Contreras, A., & Datar, F. (2015). Adoption and Impacts of Ecologically-Based Rodent Management in the Mekong Delta Region. *Journal of Environmental Science and Management*, *18*(1). |
| 1. Palis, F. G., Singleton, G. R., Brown, P. R., Huan, N. H., & Duong, N. T. (2010). Socio-cultural factors influencing the adoption of ecologically based rodent pest management. *Rodent outbreaks: Ecology and impacts*, 153. |
| 1. Ho, T. T., & Shimada, K. (2021). The effects of multiple climate change responses on economic performance of rice farms: Evidence from the Mekong Delta of Vietnam. *Journal of Cleaner Production*, *315*, 128129. |
| 1. Leon, A., & Izumi, T. (2022). Impacts of alternate wetting and drying on rice farmers’ profits and life cycle greenhouse gas emissions in An Giang Province in Vietnam. *Journal of Cleaner Production*, *354*, 131621. |
| 1. Rodriguez, D. G. P., & Nga, N. T. D. (2012). Impacts of site-specific nutrient management in irrigated rice farms in the Red River Delta, Northern Vietnam. |
| 1. Rejesus, R. M., Mutuc, M. E. M., Yasar, M., Lapitan, A. V., Palis, F. G., & Chi, T. T. N. (2012). Sending Vietnamese rice farmers back to school: Further evidence on the impacts of farmer field schools. *Canadian Journal of Agricultural Economics/Revue canadienne d'agroeconomie*, *60*(3), 407-426. |
| 1. Coxhead, I., Wattanakuljarus, A., & Nguyen, C. V. (2013). Are carbon taxes good for the poor? A general equilibrium analysis for Vietnam. *World Development*, *51*, 119-131. |
| 1. Duong, N. T., & De Groot, W. T. (2020). The impact of payment for forest environmental services (PFES) on community-level forest management in Vietnam. *Forest Policy and Economics*, *113*, 102135. |
| 1. Lampayan, R. M., Rejesus, R. M., Singleton, G. R., & Bouman, B. A. (2015). Adoption and economics of alternate wetting and drying water management for irrigated lowland rice. *Field Crops Research*, *170*, 95-108. |
| 1. Jourdain, D., Boere, E., Van den Berg, M., Dang, Q. D., Cu, T. P., Affholder, F., & Pandey, S. (2014). Water for forests to restore environmental services and alleviate poverty in Vietnam: A farm modeling approach to analyze alternative PES programs. *Land use policy*, *41*, 423-437. |
| 1. My, N. H., Demont, M., & Verbeke, W. (2021). Inclusiveness of consumer access to food safety: Evidence from certified rice in Vietnam. *Global Food Security*, *28*, 100491. |
| 1. Duong, P. B., & Thanh, P. T. (2019). Adoption and effects of modern rice varieties in Vietnam: Micro-econometric analysis of household surveys. *Economic Analysis and Policy*, *64*, 282-292. |
| 1. Hoang, V. (2021). Impact of contract farming on farmers’ income in the food value chain: A theoretical analysis and empirical study in Vietnam. *Agriculture*, *11*(8), 797. |
| 1. Do, Q. T., & Iyer, L. (2008). Land titling and rural transition in Vietnam. *Economic Development and cultural change*, *56*(3), 531-579. |
| 1. Pham, T. T., Dang, H. L., Pham, N. T. A., & Dang, H. D. (2021). Adoption of contract farming for managing agricultural risks: A case study in rice production in the Mekong Delta, Vietnam. Journal of Agribusiness in Developing and Emerging Economies. |
| 1. Ba, H. A., de Mey, Y., Thoron, S., & Demont, M. (2019). Inclusiveness of contract farming along the vertical coordination continuum: Evidence from the Vietnamese rice sector. *Land use policy*, *87*, 104050. |
| 1. Khatri-Chhetri, A., Sapkota, T. B., Sander, B. O., Arango, J., Nelson, K. M., & Wilkes, A. (2021). Financing climate change mitigation in agriculture: assessment of investment cases. *Environmental Research Letters*, *16*(12), 124044. |
| 1. Tran, H. T. M., Pham, T. D. N., & Nguyen, T. T. (2023). Education and agricultural household income: Comparative evidence from Vietnam and Thailand. *World Development Perspectives*, *29*, 100489. |
| 1. Berg, H., Lan, T. H. P., Tam, N. T., Trang, D. H., Van, P. H. T., Duc, H. N., & Da, C. T. (2023). An ecological economic comparison between integrated rice-fish farming and rice monocultures with low and high dikes in the Mekong Delta, Vietnam. *Ambio*, 1-13. |
| 1. Mills, B., Le, D. P., Ta, D. P., Nhu, L., Vo, D. T., & Labarta, R. (2023). Intensive and extensive rice farm adaptations in salinity-prone areas of the Mekong Delta. *Climate and Development*, *15*(2), 162-176. |
| 1. Ho, T. D., Tsusaka, T. W., Kuwornu, J. K., Datta, A., & Nguyen, L. T. (2022). Do rice varieties matter? Climate change adaptation and livelihood diversification among rural smallholder households in the Mekong Delta region of Vietnam. *Mitigation and Adaptation Strategies for Global Change*, *27*, 1-33. |
| 1. Ogino, A., Van Thu, N., Hosen, Y., Izumi, T., Suzuki, T., Sakai, T., ... & Kawashima, T. (2021). Environmental impacts of a rice-beef-biogas integrated system in the Mekong Delta, Vietnam evaluated by life cycle assessment. *Journal of Environmental Management*, *294*, 112900. |
| 1. Kim Dang, K., Do, T. H., Le, T. H. L., Le, T. T. H., & Pham, T. D. (2021). Impacts of farmers' adaptation to drought and salinity intrusion on rice yield in Vietnam's Mekong Delta. *Journal of Agribusiness in Developing and Emerging Economies*, *11*(1), 27-41. |
| 1. Pham, T. A. N., Loc, H. H., Tran, D. D., & Quan, N. H. (2021). The inefficiency of Vietnamese prawn-rice rotational crops: A slacks-based data envelopment analysis. *Journal of Agribusiness in Developing and Emerging Economies*, *11*(1), 60-71. |
| 1. Leigh, C., Stewart-Koster, B., Van Sang, N., Xoan, V. B., Tinh, N. T. N., Sammut, J., & Burford, M. A. (2020). Rice-shrimp ecosystems in the Mekong Delta: Linking water quality, shrimp and their natural food sources. *Science of the Total Environment*, *739*, 139931. |
| 1. Tran, N. L. D., Rañola Jr, R. F., Ole Sander, B., Reiner, W., Nguyen, D. T., & Nong, N. K. N. (2020). Determinants of adoption of climate-smart agriculture technologies in rice production in Vietnam. *International journal of climate change strategies and management*, *12*(2), 238-256. |
| 1. Kruse, J., Koch, M., Khoi, C. M., Braun, G., Sebesvari, Z., & Amelung, W. (2020). Land use change from permanent rice to alternating rice-shrimp or permanent shrimp in the coastal Mekong Delta, Vietnam: Changes in the nutrient status and binding forms. *Science of the Total Environment*, *703*, 134758. |
| 1. Paik, S., Le, D. T. P., Nhu, L. T., & Mills, B. F. (2020). Salt-tolerant rice variety adoption in the Mekong River Delta: Farmer adaptation to sea-level rise. PloS one, 15(3), e0229464. |
| 1. Keck, M., & Hung, D. T. (2019). Burn or bury? A comparative cost–benefit analysis of crop residue management practices among smallholder rice farmers in northern Vietnam. *Sustainability Science*, *14*, 375-389. |
| 1. Nguyen-Van-Hung, Tran-Van-Tuan, Meas, P., Tado, C. J. M., Kyaw, M. A., & Gummert, M. (2019). Best practices for paddy drying: case studies in Vietnam, Cambodia, Philippines, and Myanmar. *Plant Production Science*, *22*(1), 107-118. |
| 1. Thanh, D. N. C. (2019). Linking Farmers and Businesses in Integrated Organic Rice and Shrimp Farming–The Best Way for Enhancing Farmer’s Income and Sustainable Agriculture Development. *Agricultural Extension Journal*. |
| 1. Pham, N. T., & Napasintuwong, O. (2022). Farmers' Adoption and Willingness to Pay for Certified Aromatic Rice Seed in the Mekong River Delta, Vietnam. |
| 1. My, N. H., Demont, M., Van Loo, E. J., de Guia, A., Rutsaert, P., Tuan, T. H., & Verbeke, W. (2018). What is the value of sustainably-produced rice? Consumer evidence from experimental auctions in Vietnam. *Food Policy*, *79*, 283-296. |
| 1. Nguyen, V. S., Nguyen, H. M., Klotzbücher, A., Vetterlein, D., Klotzbücher, T., Jahn, R., ... & Settele, J. (2018). From science to application: field demonstrations to enhance sustainable rice production in the north of Vietnam—lessons from the LEGATO project. *Paddy and Water Environment*, *16*, 353-358. |
| 1. Berg, H., & Tam, N. T. (2018). Decreased use of pesticides for increased yields of rice and fish-options for sustainable food production in the Mekong Delta. *Science of the Total Environment*, *619*, 319-327. |
| 1. Tran, D. H., Hoang, T. N., Tokida, T., Tirol-Padre, A., & Minamikawa, K. (2018). Impacts of alternate wetting and drying on greenhouse gas emission from paddy field in Central Vietnam. *Soil science and plant nutrition*, *64*(1), 14-22. |
| 1. Le Dang, H., Li, E., Nuberg, I., & Bruwer, J. (2014). Understanding farmers’ adaptation intention to climate change: A structural equation modelling study in the Mekong Delta, Vietnam. *Environmental science & policy*, *41*, 11-22. |
| 1. Ho, T. T., & Shimada, K. (2019). The effects of climate smart agriculture and climate change adaptation on the technical efficiency of rice farming—an empirical study in the Mekong Delta of Vietnam. *Agriculture*, *9*(5), 99. |
| 1. Luu, T. D. (2020). Factors influencing farmers’ adoption of climate-smart agriculture in rice production in Vietnam’s Mekong Delta. *Asian Journal of Agriculture and Development*, *17*(1), 110-124. |
| 1. Dangc, K. K., Doan, M. T., Le, T. H. L., Nguyen, T. T. N., Pham, D. T., Do, H. T., ... & Nguyen, P. A. (2021). Assessing the Performance of Climate Smart Rice Production Systems in the Upper Part of the Vietnamese Mekong River Delta. *Asian Journal of Agriculture and Development*, *18*(1), 16-29. |
| 1. Thu, T. N., Phuong, L. B. T., Van, T. M., & Hong, S. N. (2016). Effect of water regimes and organic matter strategies on mitigating Greenhouse Gas Emission from rice cultivation and co-benefits in agriculture in Vietnam. *Int. J. Environ. Sci. Dev*, *7*, 85-90. |
| 1. Mai, V. T., Nguyen, T. D. T., Le, H. A., Richards, M. B., Sebastian, L. S., Wollenberg, E. K., ... & Sander, B. O. (2019). An investment plan for low-emission rice production in the Mekong River Delta region in support of Vietnam's Nationally Determined Contribution to the Paris Agreement. *CCAFS Working Paper*. |
| 1. Pham, V. B. (2023, March). Investing in internet of things technology: case studies of smart alternate wetting and drying irrigation. In *IOP Conference Series: Earth and Environmental Science* (Vol. 1155, No. 1, p. 012029). IOP Publishing. |
| 1. Dam, T. H. T., Tur-Cardona, J., Speelman, S., Amjath-Babu, T. S., Sam, A. S., & Zander, P. (2021). Incremental and transformative adaptation preferences of rice farmers against increasing soil salinity-Evidence from choice experiments in north central Vietnam. *Agricultural Systems*, *190*, 103090. |
| 1. Binh, N. T., Tang, L. T., Tu, N. M., Dung, T. D., & Quan, N. H. (2022). Resilience of various innovative water management practices: The case of rice production in the Vietnamese Mekong Delta floodplains. Agricultural Water Management, 270, 107739. |
| 1. Nguyen, L. (2020). Land rights and technology adoption: Improved rice varieties in Vietnam. *The Journal of Development Studies*, *56*(8), 1489-1507. |
| 1. Umetsu, C. (2022). Sustainable farming techniques and farm size for rice smallholders in the Vietnamese Mekong Delta: A slack-based technical efficiency approach. *Agriculture, Ecosystems & Environment*, *326*, 107775. |
| 1. Shrestha, S., Deb, P., & Bui, T. T. T. (2016). Adaptation strategies for rice cultivation under climate change in Central Vietnam. *Mitigation and Adaptation Strategies for Global Change*, *21*, 15-37. |
| 1. Truong An, D. (2020). Shifting crop planting calendar as a climate change adaptation solution for rice cultivation region in the Long Xuyen Quadrilateral of Vietnam. *Chilean journal of agricultural research*, *80*(4), 468-477. |
| 1. Le, T. Q. A., Shimamura, Y., & Yamada, H. (2020). Information acquisition and the adoption of a new rice variety towards the development of sustainable agriculture in rural villages in Central Vietnam. *World Development Perspectives*, *20*, 100262. |
| 1. Thanh Tam Ho, T. T. H., & Shimada, K. (2018). The impact of climate change adaptation response on rice farmers' livelihood in Soc Trang Province of Vietnam. |
| 1. Thang, T. C., Khoi, D. K., Thiep, D. H., Tinh, T. V., & Pede, V. O. (2017). Assessing the potential of climate smart agriculture in large rice field models in Vietnam. *CCAFS Working Paper*. |
| 1. Barcella, C., My, N. H. D., & Demont, M. (2018). Consumers as key drivers of change for sustainable rice production. *Rural 21: Int. J. Rural Develop*, *52*, 37-39. |
| 1. Deb, P., Tran, D. A., & Udmale, P. D. (2016). Assessment of the impacts of climate change and brackish irrigation water on rice productivity and evaluation of adaptation measures in Ca Mau province, Vietnam. *Theoretical and Applied Climatology*, *125*(3-4), 641-656. |
| 1. Mohammadi, A., Cowie, A. L., Mai, T. L. A., Brandao, M., de la Rosa, R. A., Kristiansen, P., & Joseph, S. (2017). Climate-change and health effects of using rice husk for biochar-compost: Comparing three pyrolysis systems. *Journal of Cleaner Production*, *162*, 260-272. |
| 1. Yokoyama, S., Le, C. D., Fuji, H., Fujihara, Y., & Hoshikawa, K. (2015). Economic feasibility of diversified farming system in the flood-prone Mekong Delta: Simulation for Dike Area in An Giang Province, Vietnam. *Asian Journal of Agriculture and Development*, *15*(1362-2018-4978), 21-40. |
| 1. Sivapragasam Annamalai, S. A., Chien, H. V., Li, K. S., & Duong, L. M. (2017). Pest Smart interventions and their influence on farmer pest management practices in Tra Hat village, Bac Lieu Province, Vietnam: results of a survey. |
| 1. Mohammadi, A., Cowie, A., Mai, T. L. A., de la Rosa, R. A., Kristiansen, P., Brandao, M., & Joseph, S. (2016). Biochar use for climate-change mitigation in rice cropping systems. *Journal of cleaner production*, *116*, 61-70. |
| 1. Tran, T. T. H., Zeller, M., & Suhardiman, D. (2016). Payments for ecosystem services in Hoa Binh province, Vietnam: An institutional analysis. Ecosystem Services, 22, 83-93. |
| 1. Simelton, E., & Dam, B. V. (2014). Farmers in NE Viet Nam rank values of ecosystems from seven land uses. *Ecosystem Services*, *9*, 133-138. |
| 1. Tuijnman, W., Bayrak, M. M., Hung, P. X., & Tinh, B. D. (2020). Payments for environmental services, gendered livelihoods and forest management in Vietnam: A feminist political ecology perspective. Journal of Political Ecology, 27(1), 317-334. |
| 1. Duy, V. Q. (2012). The role of access to credit in rice production efficiency of rural households in the Mekong Delta, Vietnam. *Center for Asian Studies Discussion Paper*, *284*. |
| 1. Le Ngoc, H. (2018). Contract farming effects on technical efficiency of the export-oriented rice production sector in Vietnam. |
| 1. Sato, T., Tuan, V. Q., & Beebout, S. (2014). Impact of introducing Good Agricultural Practices into the rice production in Can tho, Vietnam. In *International Annual Meeting Long Beach, CA*. |
| 1. Thuy, P. T., & Duong, P. (2022). Impacts of Payment for Forest Ecosystem Services on Local livelihoods in A Luoi District, Thua Thien Hue Province, Viet Nam. *Forest and Society*, *6*(2), 590-608. |
